# Highly Conductive Structured Catalytic Reactors for One-Step Synthesis of Dimethyl Ether

**DOI:** 10.1021/acs.iecr.0c05821

**Published:** 2021-03-09

**Authors:** Iñigo Pérez-Miqueo, Oihane Sanz, Mario Montes

**Affiliations:** Department of Applied Chemistry, Faculty of Chemistry, University of the Basque Country (UPV/EHU), Donostia-San Sebastián 20018, Spain

## Abstract

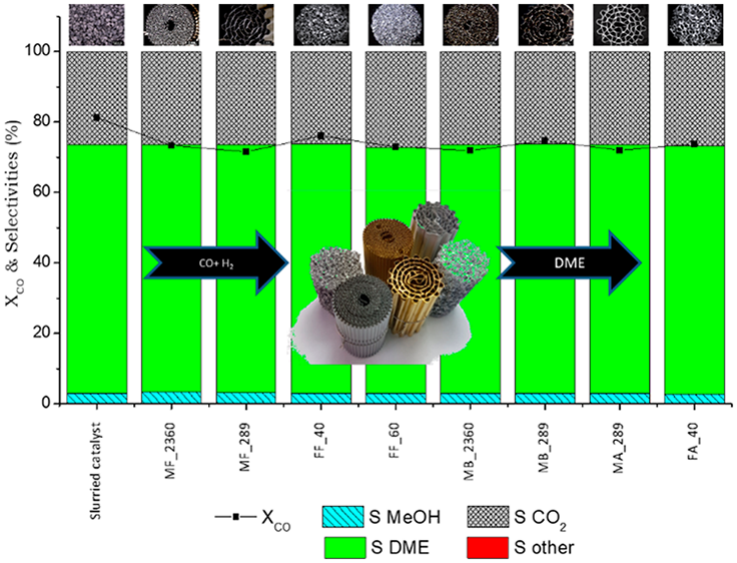

Several structured catalytic reactors for the direct synthesis of the DME reaction are compared with regard to catalyst hold-up, thermal conductivity, and volumetric productivity. Adherent and homogeneous catalyst layers were obtained by washcoating independent of the substrates’ shape and alloy. Moreover, the substrate nature (FeCrAl, brass, or aluminum) and shape (parallel cell monoliths and open foams) do not modify in great extent the CO conversion values and selectivity to the different compounds. This is reasonable since the catalytic phases are the same in all cases and the existence of mass and heat-transfer limitations was negligible in the experimental conditions studied. Structuring by washcoating exhibits less catalyst inventory per reactor volume than a packed-bed monolith. However, completely packing a monolith with powder catalyst produced a decrease in the CO conversion of around 25% with respect to the coated monolith. Moreover, by means of using the obtained highest catalyst hold-up by washcoating (0.33 gcat/cm^3^) in a brass monolith and by increasing the reaction temperature, the temperature profiles are only slightly affected. This allows to work in an almost isothermal reactor with a volumetric productivity up to 0.20 L_DME_/h·cm^3^ at 573 K.

## 1 Introduction

Dimethyl ether (DME) is known as useful intermediate in the industrial chemistry for synthesizing important chemicals such as dimethyl sulfate, methyl acetate, methyl formate, dimethoxymethane, and olefins,^1−3^ as well as propellant in agriculture, cosmetic, or painting areas.^4,5^ However, in recent years, DME has also been considered an alternative clean fuel due to its nontoxicity and reduction of emissions such as soot and NOx in its combustion. Moreover, its high cetane number (∼55–60) makes it an attractive substitute for diesel fuel.^6−8^ The European Union added DME to its potential biofuels for 2030.^9^

The conventional method of DME production consists of two steps. First, methanol is synthesized from syngas using a Cu-based catalyst, and second, the synthesized methanol is dehydrated to produce the DME over a solid acid (mainly alumina or zeolite). However, in the last decades, research on the synthesis of DME from syngas in one step, the so-called direct synthesis of DME, is acquiring relevance due to the possibility of using one reactor instead of two resulting in a simpler and more economic system, but above all, because of the reduction of the thermodynamic equilibrium limitation of the methanol synthesis, obtaining a more efficient process.^10^ Bifunctional or hybrid catalysts have been used for DME direct synthesis, with the physical mixture and the coprecipitation the most common preparation methods used.^11^ Alternatively, in recent years, advanced structures like core–shell (being the methanol synthesis catalyst the core and the solid acid the shell) has also been explored for the direct synthesis of DME.^12−15^ This catalyst structure was supposed to present the most efficient disposition of the catalyst for the reaction performance, in which the methanol is formed in the core and it is dehydrated during its flow through the shell, at the same time that the metal core is protected from deactivation by poisoning, coke deposition, or sintering.^16−18^ Therefore, this system has been reported to be very promising in enhancing the reaction yield.^19^ However, the hydrothermal conditions and reagents used to synthesize the acid catalyst shell could deactivate the methanol synthesis catalyst, especially those based on copper.^12,14,15,20^

The one-step DME reaction is a highly exothermic reaction process (Δ*H* = −246 kJ/mol) that includes methanol formation (Δ*H* = −91 kJ/mol), methanol dehydration (Δ*H* = −23 kJ/mol), and a water–gas shift reaction (Δ*H* = −41 kJ/mol). The efficient removal of heat from the reaction zone is vital because the formation of hot spots could damage the catalyst by sintering, especially in this kind of system in which Cu-based catalyst are used.^21−23^ Indeed, Azizi et al.^5^ affirm that one of the most important challenges in the synthesis of DME is to design a reactor providing maximum process intensity, advanced recovery of heat generated in the process, and preservation of catalyst activity.

Although fixed-bed reactors are the most common reactor for DME direct synthesis,^5^ due to their limited heat transfer capacity by thermal conduction,^24^ these reactors need to work at high recycling ratios to increase gas velocity and consequently heat transfer by convection and, accordingly, at low conversions.^25^ Other reactor designs have been proposed to improve the heat transfer in this reaction such as slurry phase reactors,^5^ fluidized-bed reactors,^26,27^ and structured reactors.^28,29^ None of the alternatives can be considered optimal for all cases since each of them offers advantages and disadvantages. In this sense, structured reactors such as microchannel, monolith, or foam reactors made of metal become relevant. Hayer et al.^28,29^ observed an isothermal behavior in DME direct synthesis using an integrated micro packed bed reactor–heat exchanger configuration. However, each step in the manufacturing process of microreactors should be improved to reduce the fabrication cost because the high price of this type of reactors limits their application to cases where the use of conventional technologies is impossible or when the advantages of microtechnology compensate the products costs.

Alternatively, another interesting and more economical strategy is to use monolithic reactors made of highly conductive materials such as copper,^30^ brass,^31^ and aluminum,^32^ and thus, thermal conduction through the solid matrix of the former substrate is promoted.^33^ The cell density of corrugated monoliths is another important parameter to take into consideration to obtain isothermal behavior. A higher cell density would produce a monolith with higher surface area and lower void fraction, which means a larger amount of metal and, thus, higher thermal conductivity of the systems.^32,34,35^ Furthermore, Merino et al.^32^ showed the importance of the highly effective thermal conductivity of the substrate for adequate temperature control in a highly exothermic reaction, Fischer–Tropsch synthesis (FTS), which could be achieved by using metallic monoliths of highly conductive alloys (e.g., those made of aluminum) or increasing the cell density of less conductive alloys (e.g., FeCrAl).

On the other hand, substrates with high tortuosity such as open cell foams also acquire interest in the field of structured reactors. These types of geometries generate turbulent flow in contrast to parallel cell monoliths, which present a laminar flow.^36^ Therefore, in open cell foams the contact between gas and catalyst is favored, improving mass and heat transfer.^37,38^ Indeed, Montebelli et al.^39^ studied the high potential of designing compact structured systems with open cell foams for the methanol synthesis reaction. They concluded that a reactor using this technology would improve heat transfer and would require less recycling ratios than conventional systems.

A concern with the application of structured catalytic reactors is the catalyst hold up that is typically much lower in coated systems than in conventional fixed-bed reactors reducing the productivity per reactor volume. Several authors have studied the effect of catalyst hold up of structured reactors in terms of heat and mass transfer and volumetric productivity. The maximum catalyst inventory (catalyst load per total monolith volume) obtained by the washcoating method was 0.33 g_cat_/cm^3^ for monoliths^32^ and 0.17 g_cat_/cm^3^ for foams^37^ in which a good temperature control was ensured without important internal diffusional limitations in FTS. To increase the catalyst hold up, “packed structured” reactors have been recently proposed.^40^ In such reactors, the catalyst is loaded in the form of catalyst particles randomly packed in the voids of structured substrates. In the works published by the group of Tronconi, it has been observed that the catalyst inventory can be doubled with respect to washcoated systems: 0.64 g_cat_/m^3^ using particles with around 300 μm^41^ and 0.35 g_cat_/m^3^ with particles 600 μm for foam.^42^

The aim of this work is to investigate the use of metallic structured reactors prepared with hybrid catalyst, Cu/ZnO/Al_2_O_3_ (CZA), and HZSM-5, in the direct synthesis of DME. The CZA/HZSM-5 ratio was fixed to 2, the value most cited in bibliography.^43^ The catalytic activity and the thermal behavior of monoliths and foams with different void fractions (using different cell and pore density), made of different conductive alloys and prepared with different catalyst hold-up methods (washcoating and packing), were analyzed. The above structured catalysts were also characterized by N_2_ physisorption, reactive frontal chromatography of N_2_O (RFC-N20), temperature-programmed reduction (TPR), and adherence tests.

## 2 Experimental Part

### 2.1 Metallic Substrates

In-house-fabricated metallic monoliths of different alloys [FeCrAl (Fecralloy, Goodfellow), brass (Cu63Zn37, Goodfellow), and aluminum (>99 wt%, INASA)] were prepared by rolling up corrugated and flat metallic foils. Two different metallic foams were used: FeCrAl (BRC2005-08 Metpore) and aluminum (6061, Duocel) were provided by Selee Co., and ERG Aerospace, respectively. The geometric parameters of the used structured substrates are summarized in Table S1.

**Figure 1:**
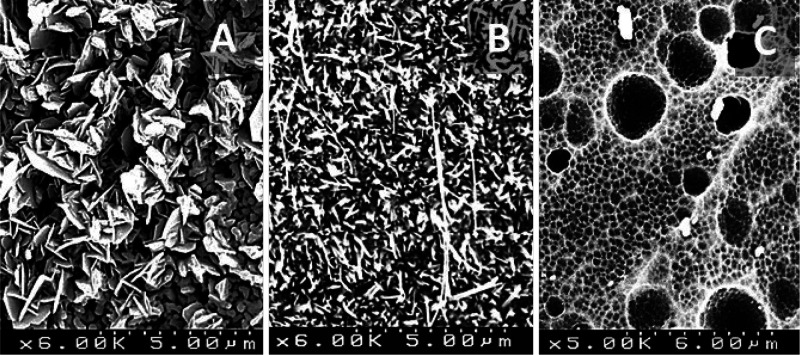
SEM micrographs of metal substrates after pretreatment: (A) FeCrAl, (B) brass, and (C) aluminum.

The structured substrates samples were cleaned with water and soap followed by acetone rinsing. Then, the substrates were treated to obtain adequate surface roughness. FeCrAl alloy substrates were calcined under air atmosphere at 1173 K (10 K/min) for 22 h^44^ (Figure 1A). Brass monoliths were calcined at 823 K (10 K/min) during 18 h under air atmosphere^45^ (Figure 1B). The aluminum pretreatment is done with dishwasher detergent (Calgonit Powerball All in One by Reckitt Benckiser) at 70 °C for 40 min. The pH produced by the detergent mixture (pH ≈ 10.5 at 1% concentration) due to the presence of sodium carbonate (20–30%), the main detergent builder, attacks aluminum due to its amphoteric character. This attack is enhanced by the strong oxidizing character of sodium percarbonate (5–10%) used as bleaching agent. We believe that other minor components of the complex mixture of the commercial product could also play an important role in the attack on aluminum, but it is difficult to contrast this given the secrecy of the exact formulation kept by the manufacturers of this type of product. Nearly the only source of information on its composition is the limited information available in the Material Safety Data Sheet (MSDS). Finally, the important point is that the pitting attack produced by this simple pretreatment creates a rough surface of the aluminum that favors the adherence of the subsequent catalytic coating on the aluminum surface (Figure 1C). Finally, the aluminum substrates were rinsed with water, dried at 393 K during 30 min, and calcined at 773 K (10 K/min) for 2 h under air atmosphere.

### 2.2 Structured Catalyst Preparation

Structured catalytic reactors were prepared by the washcoating method. For this purpose, a preliminary study, not shown here, on the variables that control the washcoating process was carried out.^46^ The coating characteristics (specific load, homogeneity, and adhesion) were analyzed to choose the best recipe. A hybrid catalyst slurry was prepared by mixing 11.9% of the presynthesized Cu/ZnO/Al_2_O_3_ catalyst (CZA), 6.0% of ZSM-5 zeolite, 1.3% of colloidal Al_2_O_3_ (Nyacol AL20), 0.8% of poly(vinyl alcohol) (PVA) (Mowiol 4-88), and 80% of deionized water.

CuO/ZnO/Al_2_O_3_ catalyst was synthesized by conventional coprecipitation method.^47^ A 1 M solution of different metal precursors [Cu(NO_3_)_2_·3H_2_O, Zn(NO_3_)_2_·6H_2_O and Al(NO_3_)_3_·9H_2_O provided by Sigma-Aldrich, with a 6:3:1 molar ratio] was pumped (at constant flow rate of 5 mL/min) into a vessel containing a starting volume of 200 mL of distilled water at 343 K. During this process, a solution of 1 M Na_2_CO_3_ (Panreac) was used as precipitant and was pumped simultaneously to adjust the pH to 7. After aging, the precipitate was filtered and was washed with plenty of distilled water. Finally, it was dried at 373 K overnight.

ZSM-5 zeolite provided by Zeolyst International was calcined at 773 K (2 K/min) for 5 h.

During the washcoating process, structured substrates were dipped into the catalyst slurry at a constant speed of 3 cm/min, remained dipped for 1 min, and withdrawn at the same speed. Then excess slurry was removed by centrifugation (400 rpm, 1 min). Immediately, the structured catalysts were dried at 393 K for 30 min, and the procedure was repeated until the desired amount of catalyst was coated. Finally, the structured catalysts were calcined at 673 K (2 K/min) for 3 h.

Additionally, an aliquot of the catalyst slurry was dried and calcined under the same conditions as those used to prepare monolithic catalyst to obtain the slurried catalyst. This sample is representative of the solid layer coating the surface of structured substrate, exhibiting a similar composition and thermal history, and will be called slurried catalyst.

Packed monoliths were prepared by filling 289 cpsi brass monoliths with slurried catalyst particles of 300–500 μm. Two different packed monoliths were prepared:

- 3 g of catalyst: the monolith was completely packed with the slurried catalyst’s particles.
- 1 g of catalyst: the monolith was packed with a mixture of the slurried catalyst’s particles and SiC particles (Carborondum, 500 μm) with a ratio catalyst/SiC of 1:3.

Samples are referred to as AB_C_DE, where A is the type of structured substrate (M for monolith and F for foam), B is the substrate alloy (A for aluminum, F for FeCrAl and B for brass), C is the cell or pore density of the structured substrate (289 or 2360 cpsi and 40 or 60 ppi), D is the hold-up method (W for washcoating and P for packing), and E is the nominal amount of catalyst (g).

### 2.3 Characterization

Textural properties of structured and slurried catalysts were obtained by N_2_ physisorption in a Micromeritics ASAP 2020. For a structured catalyst, a cell allowing analysis of entire samples was used. Samples were previously degassed at 180 °C up to a vacuum level of 10 μmHg for 8 h and finally analyzed at 77 K. The specific surface area was calculated with the BET equation, and the total pore volume (*V*) was determined at 0.99*P*/*P*_0_. The equivalent pore diameter used was 4 V/*S*_BET_.

The copper metallic surface area was measured by reactive frontal chromatography of N_2_O (RFC-N_2_O) employing a Micromeritics AutoChem II 2920. The catalyst, which was previously reduced with a flow of 10% H_2_/Ar, was submitted to pulses of N_2_O at 333 K in He flow. At this temperature surface copper was oxidized to Cu_2_O^48^
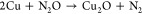
With a cool trap of a mixture of liquid N_2_ and acetone, the N_2_O was trapped and the amount of produced N_2_ was quantified with a TCD detector (*n*_N_2__ = moles of N_2_). Being 0.068 nm^2^ the atomic cross-sectional area of copper (*d*_Cu_), the Cu metallic surface area was calculated as
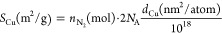
Temperature-programmed reduction (H_2_-TPR) was carried out in a Micromeritics AutoChem II 2920. A 10% H_2_/Ar mixture was flown through the sample in the range of 313–1173 K with a 10 K/min heating rate. H_2_ consumption was measured with a TCD. The structured substrates without catalyst coating did not present noticeable reducibility by themselves. Several measurements of coated structured substrates have been done, with experimental error around 10%. The limited accuracy of this calculation may be related to the fact that the Cu content of the catalyst used is the nominal content corresponding to the slurry formulation. The precise analysis of said Cu content is complex, and above all it is a destructive test since it should be carried out with the whole monolith coated.

The adherence of the catalytic layer deposited on the substrates was measured by weight loss caused during sonication of the coated monolith immersed in petroleum ether (OPPAC) during 30 min at room temperature.^49^

The surface morphology of the structured substrates after pretreatment was observed by SEM (Hitachi S-2700).

### 2.4 Catalytic Test

**Figure 2:**
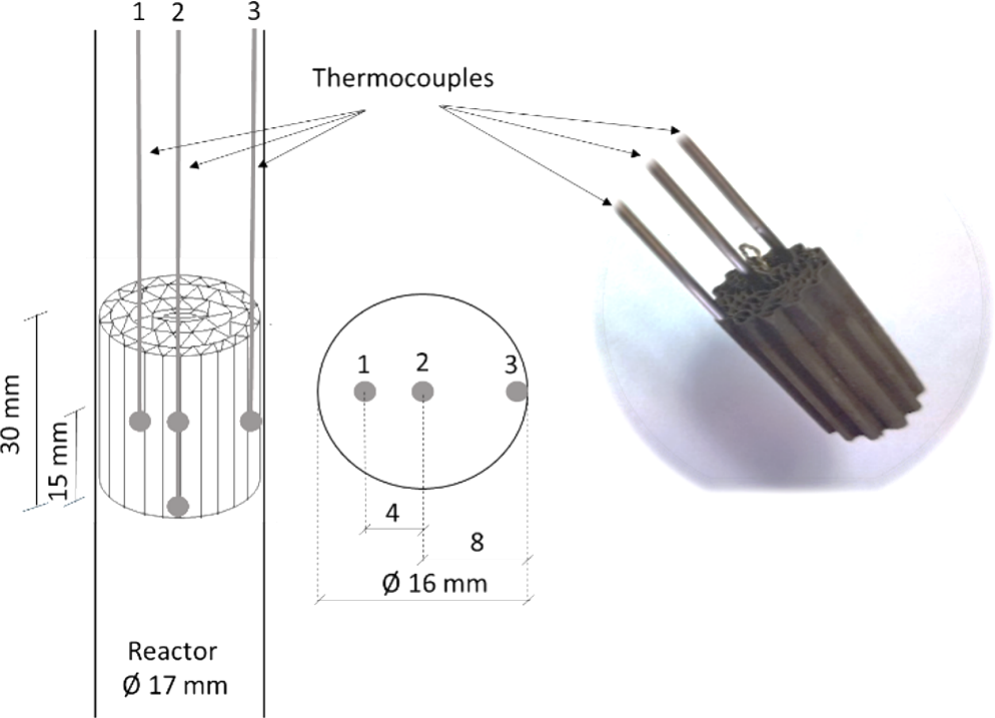
Scheme of the structured catalytic reactor with thermocouple positions.

Direct synthesis of DME reaction was carried out in a Microactivity Reference lab reactor (PID Eng&Tech). The structured catalysts were placed inside a Hastelloy tubular reactor with an inner diameter of 17 mm. The reaction temperature was monitored by three thermocouples set in three radial positions as shown in Figure 2. Before reaction, the catalyst was reduced at 518 K during 4 h (2 K/min) with 5%H_2_ in N_2_ at atmospheric pressure. The reaction was carried out at 533 K and 4 MPa and was fed with a mixture of 90% of syngas (H_2_/CO = 2) in N_2_ with a WHSV of 1.7–6.8 L_syngas_/g_cat_·h (being the catalyst a mixture of CZA+HZSM-5). The products were taken out through thermostatic line and were analyzed by GC (Agilent 7890A) using TCD (HP-PLOT/Q and HP-MOLESIEVE) and FID (HP-PLOT/Q) detectors.

The conversion was defined as the ratio of the amount of CO converted to the amount of CO fed to the reactor and was expressed in molar %. The selectivity (molar %) to each product was defined as the ratio of carbon moles in a specific product to the moles of CO converted.

The volumetric heat duty (*Q*) is calculated according eq 1
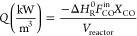
1where Δ*H*_R_^0^ is the standard reaction enthalpy set to −82 kJ/mol_CO_ and *V*_reactor_ is the volume occupied by the structured catalyst.

The absence of internal temperature gradients inside the catalyst particle in a packed monolith can be assumed because the following criterion (eq 2), which depends on the dimensionless activation energy (γ), the internal Prater number (β_i_), and the Wheeler–Weisz modulus (ηφ^2^), is satisfied^50^
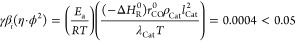
2where *T* is the reaction temperature (523 K), *R* is the gas constant (8.314 J/mol/K), *E*_a_ is the apparent activation energy (85 kJ/mol) obtained from the bibliography,^51^ Δ*r*_CO_^0^ is the CO consumption rate (6.61 × 10^–6^ mol/s/g_Cat,_ mol CO feed × CO_eq_ conversion at steady sate), Δ*H*_R_^0^ is the is the standard reaction enthalpy (−82 kJ/mol_CO_), *ρ*_Cat_ is the catalyst particle density (0.912 × 10^6^ g/m^3^), *l*_Cat_ is the characteristics catalyst length (*D*_pellet_/6 = 83 × 10^–6^ m), and *λ*_Cat_ is the catalyst thermal conductivity (0.3 W/m/K).

The absence of external interphase (gas–solid) heat transport limitations can be assumed due to the fact the Mears criterion (eq 3) is satisfied^52^
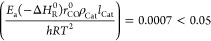
3where *T* is the reaction temperature (523 K), *R* is the gas constant (8.314 J/mol/K), *E*_a_ is the apparent activation energy (85 kJ/mol), Δ*r*_CO_^0^ is the consumption rate (6.61 × 10^–6^ mol/s/g_Cat_), Δ*H*_R_^0^ is the is the standard reaction enthalpy (−82 kJ/mol_CO_), *ρ*_Cat_ is the catalyst particle density (0.989 × 10^6^ g/m^3^), and *l*_Cat_ is the characteristics catalyst length (*D*_pellet_/6 = 83 × 10^–6^ m). The gas–solid heat transfer coefficient, *h* (2441 W/m^2^/K), is found from the Nussel number (, where λ is the thermal conductivity of the gas phase (0.11W/m/K)) and Nu is estimated with a packed bed correlation based on bed porosity (, with *ε*_PB_ = 0.38).^50^

## 3 Results

### 3.1 Washcoated Structured Reactors: Effect of Substrate Nature and Shape

As shown in Table S2, a series of structured reactors with the same catalyst loading (∼1 g/structured catalyst) made of different alloys (FeCrAl, aluminum and brass) and different geometries (parallel channel monoliths and interconnected pore foam) were prepared. The properties of the washcoating (number of coatings applied, layer thickness and catalyst coating adherence), the textural properties, copper metal surface area, and reducibility are also compiled in Table S2.

**Figure 3:**
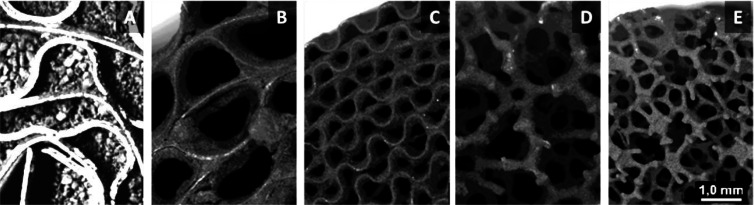
Image of structured catalytic reactors: (A) MB_289_P3, (B) MF_289_W1, (C) MF_2360_W1, (G) FF_40_W1, and (E) FF_60 W1 (all images are at the same scale).

All structured substrates were washcoated, and the obtained coatings were homogeneous and adherent, without plugging the monolith’s channels or the foam’s pores (Table S2 and Figure 3). When an attempt was made to load a higher amount of catalyst, especially in the case of foams, coating problems were observed (pore clogging, less adherence...). Therefore, it was decided to compare all structured substrates loaded with 1 g.

The alloy used for structured catalyst preparation did not affect in great extent the loading process (Table S2), but it can be observed that the catalyst load depends on the monolith’s cell density: the higher the geometric surface area (Table S1) the lower the number of coatings needed for the same total loading. In addition, foams presented a higher specific load than the parallel channel monolith due to geometry difference with respect to monoliths:^37^ foams present large tortuosity where accumulations could be produced (Figure 3).

The textural and reducibility properties of the catalyst coatings were similar to those of the slurried catalyst (Table S2). Only the structured catalyst produced a slight decrease in the Cu surface area (a decrease of ∼10%) in comparison to that of the slurried catalyst.

Regarding catalytic activity, it can be observed that the different structured catalyst prepared presented a slight decrease in the CO conversion values in comparison to that of the slurried catalysts (Table S3), probably due to slight decrease observed (∼10%) in the copper surface area of the structured catalyst (Table S2). However, it was noticeable that all structured catalysts produced similar CO conversion and selectivity values and isothermal temperature profile (axial and radial) between them at the same reaction conditions, independent of substrate’s shape and alloy (Table S3).

Moreover, the reaction was also carried out at different space velocities (Table S3). As expected, the CO conversion decreased when the space velocity was increased. However, as previously stated, the CO conversion and selectivity to different compounds remained almost similar for all structured catalyst.

### 3.2 Process Intensification

**Figure 4:**
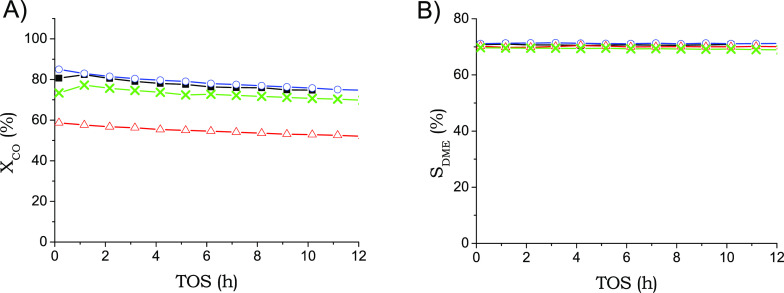
CO conversion (A) and DME selectivity (B) obtained at 533 K, 4 MPa and 1.7 L_syngas_/g_cat_·h for different brass monoliths: ■, MB_289_W1, o MB_289_W2; X, MB_289_P1; Δ, MB_289_P3.

On the other hand, the intensification of the DME volumetric productivity was evaluated. First, the effect of the catalyst loading on coated and packed 289 cpsi brass monoliths was studied (Figure 4). The results showed that increasing the catalyst loading by washcoating from 1 g (MB_289_W1) to 2 g (MB_289_W2) did not modify the CO conversion values nor selectivities, so double DME volumetric productivity (from 0.050 to 0.100 L_DME_/h·cm^3^) was achieved .

On the other hand, in order to increase the catalyst hold up, 289 cpsi brass monoliths were also filled with the slurried catalyst (dry and calcined suspension sieved between 300 and 500 μm). Two types of filling were made: monolith completely packed with the hybrid catalyst’s particles (MB_289_P3) and monolith packed with a mixture of the hybrid catalyst’s particles and SiC particles (MB_289_P1). The results in Figure 4A show a decrease in CO conversion when the monolith is fully filled with catalyst’s particles (3 g cat) with respect to the coated monoliths. However, the selectivities obtained were similar for the two catalyst incorporation methods (Figure 4B). This results in a volumetric productivity of MB_289_P3 similar to that of the monolith coated with 2 g (0.105 L_DME_/h·cm^3^). However, when filling with 1 g of hybrid catalyst diluted with SiC, the same CO conversion and selectivity were observed as in the coated monoliths, being the volumetric productivity similar to that of the coated monolith with the same amount of catalyst (Figure 4).

The radial and axial temperature profiles of the coated and packed monoliths were also measured, and the results did not show relevant changes in the profiles, being able to assume an isothermal system (results not shown).

**Figure 5:**
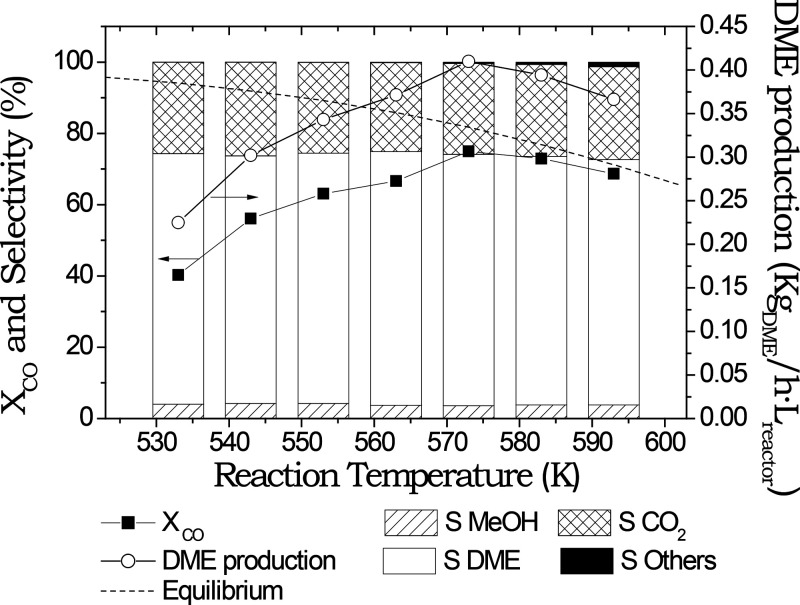
CO conversion, selectivity, and DME production for direct synthesis of DME with a brass monolith coated with 2 g of catalyst at 4 MPa and 3.4 L_syngas_/g_cat_·h with different reaction temperatures.

The MB_289_W2 sample with a catalyst inventory of 0.33 g_Cat_/cm^3^ was selected to study the catalytic behavior as a function of reaction temperature (533–593 K) at a constant space velocity of 3.4 L_syngas_/g_cat_·h. Seven independent experiments were carried out with a freshly filled monolith in each experiment. The CO conversion and selectivity to different compounds were measured at 10 h on stream when the values were stable. The results from Figure 5 showed an increase in the CO conversion values when the reaction temperatures increased until 573 K. From that temperature, the CO conversion decreased. With this study, it could also observed that the DME volumetric productivity behaved in the same way, reaching the maximum production at 573 K.

**Figure 6:**
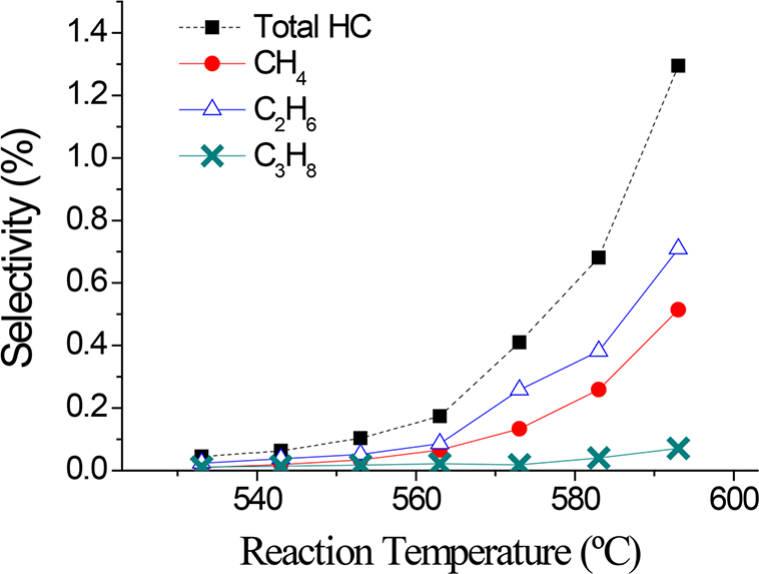
Selectivity of light hydrocarbons produced in the direct synthesis of DME in a brass monolith with 2 g of catalysts at different reaction temperature. Reaction conditions: 4 MPa and 3.4 L_syngas_/g_cat_·h.

On the other hand, the increase in the reaction temperature produced an increase in the selectivity to other compounds (Figures 5 and 6). The selectivity of other compounds synthesized in the reaction conditions (mainly light hydrocarbons) exponentially increases from 573 K (Figure 6).

**Figure 7:**
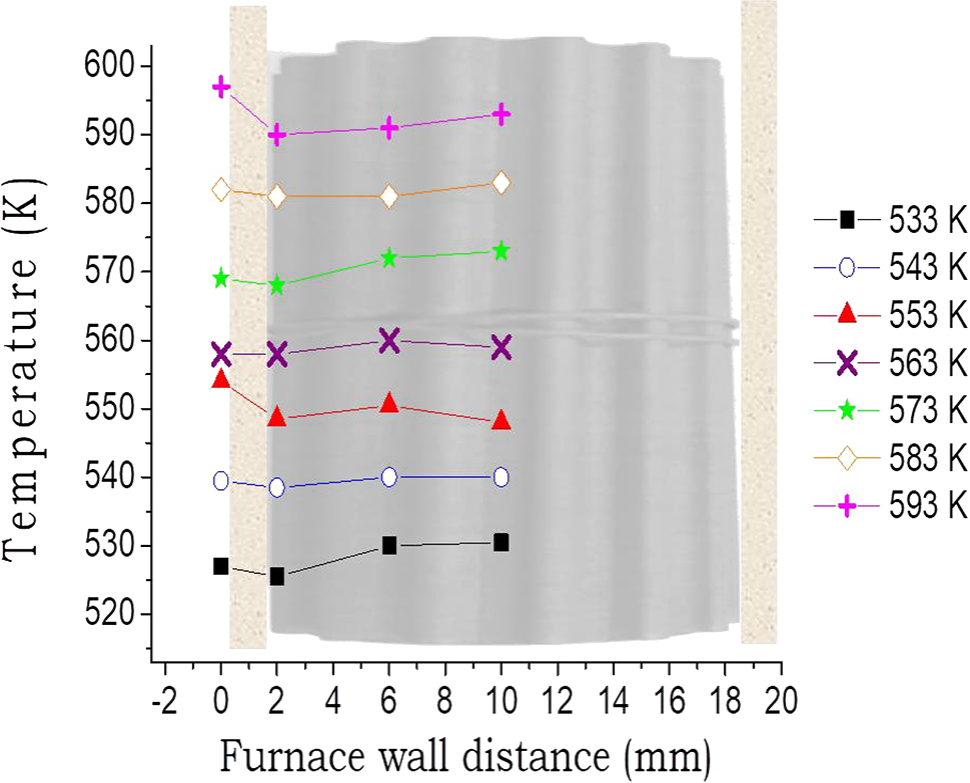
Radial temperature profile of the 2 g brass monoliths in the direct synthesis of DME at different temperatures. Reaction conditions: 4 MPa and 3.4 L_syngas_/g_cat_·h.

Finally, in the experiments with different reaction temperature the radial temperatures of the monoliths were also monitored (Figure 7). The results showed that the radial profile was almost flat in all of the experiments independent of the reaction temperature.

## 4 Discussion

To study the heat dissipation capacity of metallic structured substrates, alloys of different thermal conductivity such as steel, brass, and aluminum have been selected (Table S1). Different geometries of the substrates are also compared, monoliths with parallel longitudinal channels and open-cell foams, which produce notable differences in the flow pattern, much more turbulent in the foams. After the specific treatment of these substrates (see Experimental Part, Figure 1), a rough surface layer is obtained that is chemically compatible with the deposited catalyst. In the case of FeCrAl and aluminum, a layer of alumina is formed, and in the case of brass, a layer of ZnO. In this way, both chemical (compatibility, ability to form chemical bonds) and mechanics adhesion (anchoring in roughness) are enhanced by the metal substrate pretreatments.

The washcoating process using a catalyst particle suspension is an excellent method to coat metallic structured substrates for different applications.^31,32,35,37,38^ The results obtained is this work show that the slurry formulation of hybrid catalyst is perfectly suited to all of the materials and shapes of the structured substrate used. This method produces excellent coatings taking into account the catalytic coatings adherence and homogeneity (without accumulations and channel’s plugging) and preservation of the catalyst properties during deposition on structured substrate (Tables S2 and S3, Figure 3). The adherence of catalytic coating was measured taking into account the percentage of catalytic layer remaining adhered after the ultrasonic tests.^49^ There are different ways to determine the adherence on coated structured substrates being the ultrasonic test the most demanding method that is shown in the literature.^53^ The obtained adherence values are in agreement with those of similar washcoated structured substrates.^31,32,35,37,38^ Moreover, the used slurry formulation of hybrid catalyst preserved its activity. The type of contact between the two catalysts (CZA and zeolite) can influence their deactivation/stability as observed by García-Trenco et al.^54^ They observed that preparing the hybrid catalyst by physical mixture is better than by slurry. They attributed this deactivation occurs because during the slurry preparation part of the mixed oxides, the active phase of CZA, suffers hydration forming a hydrotalcite phase that is not active in this reaction. However, in our previous work,^46^ we have observed that calcining CZA/zeolite slurry, hydrotalcite is not formed and the deactivation of CZA is avoided.

The CO conversions obtained for all structured catalyst prepared by washcoating were very similar (Table S3). By varying the type of structured system, different modifications in reactor properties have been made: catalytic layer thickness, flow pattern (monolith vs foam), and heat removal capacity. In this work, different catalyst layer were achieved (∼20–100 μm, Table S2) with the same catalyst hold-up (∼0.167 g_Cat_/cm^3^), due to the use of different structured substrates presenting different geometric surface area (Table S1). However, there were no significant changes in the one-step synthesis of dimethyl ether with different thicknesses (Table S3). Hence, no diffusional limitations were appreciated, which agrees with previous studies. Indeed, the highest thickness achieved, 100 μm, is equivalent from the point of view of diffusion limitations to a spherical particle of 600 μm,^55^ the catalyst particle size used in one-step synthesis of dimethyl ether in some studies that neither observed diffusion problems.^1,56,57^

On the other hand, the change of the substrate shape from parallel cell monoliths to open cell foams did not generate relevant changes in activity at the studied conditions (Table S3). Open cell foams are characterized by their tortuosity, which generates improvement in mass and heat transfer in comparison to parallel channels.^36,38^ Consequently, open cell foams are considered a good alternative for reactions with rapid kinetics and prone to suffer from diffusional limitations.^58^ In reactions such as methanol steam reforming,^31^ oxidation of toluene,^59^ Fischer–Trospch synthesis^37^ or selective hydrogenation of 1,3-butadiene^38^ it was observed the improvement when using foams instead of monoliths. Nevertheless, the direct synthesis of DME did not show that behavior under the studied conditions, which suggests that there is no external diffusion control under the conditions used.

The high conductivity of metallic structured substrate in comparison to powder catalysts make them interesting for high exothermic or endothermic reactions. While in a conventional fixed bed reactor the heat transfer is controlled by convection, in metallic structured reactors, the conduction through the material is also relevant for the heat transfer phenomenon. Therefore, the higher the conductivity of the substrate used, the greater the heat transfer rate. In our work, different parallel cell monoliths and foams (high 2360 cpsi and low 289 cpsi) as well as different alloys with different heat dissipation capacity were used (Table S1). Very small temperature gradients (axial and radial) inside structured reactors are obtained at 533 K and 0.167 g_Cat_/cm^3^ (Table S3). The volumetric heat duty (*Q*) calculated in the experiments in all structured catalyst was lower than 120 kW/m^3^ (Table S3), being a moderate value to produce significant differences in the temperature profile. In our previous works,^32,37^ using similar structured reactors for FTS we observed that temperature differences were important when using low conductive material (FeCrAl) when *Q* is higher than 160 kW/m^3^.

Once the good results of the structured catalysts prepared were observed, we tried to intensify the process by increasing the volumetric productivity of the 289 cpsi brass monoliths by washcoating and packing. By increasing the catalyst loading in the 6 cm^3^ monolith, we can increase the productivity per volume of reactor.

Doubling the catalyst loading by washcoating means doubling the catalyst layer thickness, which might produce mass transfer limitations. However, after coating a monolith with 2 g (0.33 g/cm^3^) of catalyst (∼100 μm of catalyst layer thickness) the result showed similar CO conversion and selectivity to different products than those of the structured catalyst with 1 g (0.17 g/cm^3^) (Figure 4 and Table S3). In this way, the volumetric productivity of DME was increased from 0.050 to 0.100 L_DME_/h·cm^3^.

The catalyst inventory (0.33 g/cm^3^) is the maximum possible over these substrates, since higher loads produce heterogeneity problems with channel obstruction and loss of adhesion. To increase the catalyst hold up, packed brass monoliths were prepared by loading slurried catalyst particles in the voids of structured substrate. This has allowed the catalyst inventory to be increased up to 0.50 g/cm^3^ with particles of 300–500 μm.

As it could be observed, the fact of completely packing a monolith with 3 g of catalyst produced a decrease in the CO conversion of around 25% with respect to the coated monolith (Figure 4). This lower activity is not due to changes in the catalyst since, as previously seen, the copper metallic surface area is not altered (Table S2).

Another possible cause of this behavior could be due to a deactivation of the methanol synthesis catalyst and/or methanol dehydration catalyst. The group of Martínez deeply study the deactivation of the CZA/zeolite system and determined that the loss of activity during the direct synthesis of DME is due to a gradual decrease in the methanol synthesis catalyst activity more than to a deactivation of the zeolite.^54,60^ The exothermicity of the reaction could generate temperature peaks that deactivate the catalyst by copper sintering. However, the CO conversion versus time curves did not either show a different behavior (Figure 4). In addition, due to the small value of the internal Prater number^50^ and the absence of gas–solid limitations verified by applying the appropriate Mear’s criterion^52^ (see the Experimental Part) no significant temperature gradients are to be expected within the catalyst particles. Therefore, we could rule out the thermal sintering of Cu as the cause of the lower activity of the monolith filled with 3 g of catalyst. Regarding the deactivation of zeolite, Bobadilla et al.^61^ observed that the deactivation due to coke formation in glycerol reforming could be different in coated monoliths that in catalyst pellets, due to the blockage produced by coke between the catalyst pellets in the fixed bed, while the wide space in the monolith channels allows avoiding said plugging. Further experiments such as a deactivation (formation of coke) study would be helpful to explain this unexpected result.

On the other hand, the increase of the catalyst load in the structured systems (for the same space velocity) implies a proportional increase of the gas feed flow rate. This increase in flow could generate a decrease in the temperature of the gases at the inlet, as cited by certain authors, due to a shorter residence time in the gas preheating system, generating an axial profile.^62−64^ However, the axial profile of the system also depends on the exothermicity of the reaction. Fratalocchi et al.^65^ studied the axial and radial profile of a packed-bed foam on the Fischer–Tropsch synthesis (exothermic reaction). Their results showed a radial profile of the system due to a lower heat input from the furnace. Furthermore, this lower energy input from the furnace causes that the gases fed to enter at a lower temperature, which also caused an axial profile to be generated along the foam.

In our study, an additional experiment was done by reducing the catalyst load in the packed monoliths: 1 g of catalyst is diluted in SiC. Therefore, the same flow rate as in the monolith coated with 1 g of catalyst is fed and the conductivity of the “mini-beds” is promoted by diluting the catalyst with SiC. The catalytic test showed that this packed monolith recovered the conversion of CO to similar values than that of the coated one (Figure 4). However, the measurements done on both packed monoliths did not show noticeable changes in the radial profiles with a variation of 2 °C regardless of the load used. Similarly, no gradients were observed in the axial profile (between the control point located at the exit of the monolith and the measurement point located halfway up the monolith) that could justify this change in activity.

The possibility that the loss of activity of the monolith filled with 3 g of catalyst was due to problems of heterogeneity in the flow in the microbeds of each channel was also considered. This heterogeneity could be due to deficiencies in the filling process or even to heterogeneities in the size of the microchannels given the artisan character of their homemade construction. To rule out these eventual problems, the experiment was repeated three times with three different monoliths and, therefore, three catalyst filling processes. The results confirmed a loss of activity of around 20–25% in all cases. Regarding the positive effect on the flow that the dilution with SiC could have, we believe it is unlikely since special attention was paid, always using SiC of the same particle size as the catalyst.

The analysis of all these results on the intensification of the process by increasing the catalyst inventory does not produce a clear conclusion to explain the loss of productivity when the monolith is packed with 3 g of catalyst particles. It appears that this loss of productivity is not due to the sintering of Cu due to poor temperature control nor are there significant radial or axial temperature gradients that suggest differences due to a lack of bed isothermicity. Therefore, it will be necessary to delve into this problem in the near future to find a satisfactory explanation.

On the other hand, volumetric productivity could also be increased by changing reaction conditions using coated brass monolith. As it is observed in the Table S3, space velocities of 3.4 L_syngas_/g_cat_·h allow for producing CO conversion values low enough to increase afterward them by means of increasing the reaction temperature, favoring the reaction kinetic.

The increase in the reaction temperature did not modify the DME selectivity to a great extent, which agrees with the equilibrium data of the reaction (Figure S1), but the CO conversion increased with temperature until 573 K and consequently the DME productivity, reaching to 0.2 L_DME_/h·cm^3^ (Figure 5). However, from 573 K the CO conversion values start decreasing due to thermodynamic equilibrium of the reaction favored at low temperatures.^66^

To our knowledge, there are few reports about direct synthesis of DME in structured reactors. Hu et al.^67^ and the research group of Venvik in collaboration with the Karlsruhe Institute of Technology (KIT)^28,51,68^ studied the direct synthesis of DME in microchannel reactors, studying the effect of different parameters such as reaction temperature, pressure, space velocity, etc. On the other hand, other promising advanced catalysts such as the core–shell systems had been also studied.^12−15^ Unfortunately, the lack of standard reaction conditions and different methanol synthesis/dehydration catalyst ratios makes difficult a comparison with the literature. In addition to that, the volumetric productivity comparison could not be done because of the lack of some important data such as catalyst mass loaded in the reactor, volume of the reactor, etc. For example, Hayer et al.^68^ with a physical mixture of a CZA/Al_2_O_3_ (ratio of 1:1) system in a microchannel reactor at 533 K, 5 MPa, and 4.5 L_syn_/g_cat_·h were obtained around 30% and 72% of CO conversion and DME selectivity, respectively, while in this work with the reaction conditions of 533 K, 4 MPa, and 3.4 L_syn_/g_cat_·h the obtained values were around 40 and 70%, respectively, results that can be considered comparable.

In addition, the preparation of core–shell catalysts can be challenging due to the damages produced by the synthesis of the second catalyst on the previously deposited^12,14,15^ or because of diffusion limitations due to the core thickness.^19^ Indeed, Baracchini et al.^19^ obtained around 1% of conversion at 533 K, 4.5 MPa, and 6.75 L_syn_/g_cat_·h with a CZA@HZSM-5 core–shell catalyst. However, other authors, such as Wang et al.,^14^ prepared a CZA@SiO_2_–Al_2_O_3_ core–shell catalyst obtaining a CO conversion and DME selectivity of 71.1 and 61.9%, respectively, at 533 K, 5 MPa, and 1.5 L_syn_/g_cat_·h. This last result is similar to that obtained in this work, in which the CO conversion and DME selectivity was around 72 and 70%, respectively, at the same reaction temperature, 4 MPa and 1.7 L_syn_/g_cat_·h.

Moreover, on increasing the catalyst hold-up to 0.33 g_Cat_/cm^3^ and increasing reaction temperature using brass monolith, the temperature differences are slightly affected (Figure 7), although the volumetric heat duty reached 280 kW/m^3^ at 573 K. Brass is a highly conductive metal that allows excellent temperature control. Similar behavior was observed using aluminum monoliths in FTS obtaining flat temperature profile when *Q* was as high as 630 kW/m^3^,.^32^

Nevertheless, the increase in the reaction temperature also produced a slight increase in the selectivity to byproducts (mainly light hydrocarbons) (Figures 5 and 6). Methane is one of the produced compounds, which could suggest the methanation of CO/CO_2_.^69,70^ However, formation other hydrocarbon such as ethylene and acetylene started to increase with the temperature. The dehydration of methanol to hydrocarbons is promoted at high temperatures,^54,60^ especially at temperatures above 573 K.^60,71^ In addition, the exponential growth of hydrocarbon selectivity from 573 K is shown in Figure 6.

## 5 Conclusion

Structured catalysts for direct synthesis of DME were prepared successfully by a washcoating method in different substrates. Adherent and homogeneous coatings were obtained independent of the substrates’ shape and alloy, all above 80% adherence.

Moreover, the substrate nature (FeCrAl, brass, or aluminum) and shape (parallel cell monoliths and open foams) do not modify in great extent the CO conversion values and selectivity to the different compounds. This is reasonable since the catalytic phases are the same in all cases and the existence of mass and heat transfer limitations was negligible in the experimental conditions studied. No heat transfer limitations were observed because the volumetric heat duty produced was not high enough to cause significant temperature differences using metallic structured reactors.

As a consequence, due to the excellent behavior of metallic substrates, the volumetric productivity of 289 cpsi brass monoliths was increased (process intensification) by varying the catalyst loading and reaction conditions. The DME volumetric productivity can be increased four times by means of doubling the volumetric loading on the monolith and reaction temperature. Above 573 K, the approach to the thermodynamic equilibrium and the excess dehydration of DME to hydrocarbons is favored. The high conductivity of brass monoliths produced almost isothermal systems in all of the experiments carried out.

## Supporting Information Available

Table S1, main characteristics of the used structured substrates; Table S2, comparison of physicochemical properties of structured catalysts; Table S3, comparison of catalytic activity of structured catalyst; Figure S1, CO conversion and selectivities for direct synthesis of DME in the equilibrium at different temperatures (PDF)

## Nomenclature

*E*_a_: apparent activation energy
D_pellet_: pellet diameter
*h*: gas–solid heat transfer coefficient
K_e,a_: axial effective thermal conductivity
K_r,a_: radial effective thermal conductivity
*l*_Cat_: characteristics catalyst length
Nu: Nussel number
*R*: gas constant
Δ*r*_CO_^0^: CO consumption rate
*T*: reaction temperature
*V*_reactor_: volume occupied by the structured catalyst

## Greek Letters

β_i_: internal Prater number
γ: dimensionless activation energy
Δ*H*_R_^0^: standard reaction enthalpy
*λ*_Cat_: catalyst thermal conductivity
λ: thermal conductivity of the gas phase
*ρ*_Cat_: catalyst particle density
ηφ^2^: Wheeler–Weisz modulus
