# Supplementary material for: Highly Conductive Structured Catalytic Reactors for One-Step Synthesis of Dimethyl Ether
Source: Ind Eng Chem Res. 2021 Mar 9;60(18):6676–86. doi: 10.1021/acs.iecr.0c05821 (PMC8924795; doi:10.1021/acs.iecr.0c05821)
Supplement: Supplementary file 1 — ie0c05821_si_001.pdf [file ie0c05821_si_001.pdf]

Supporting information

Highly conductive structured catalytic reactors for one-step synthesis of dimethyl ether

Iñigo Pérez-Miqueo, Oihane Sanz, Mario Montes*

Dept. Applied Chemistry, Faculty of Chemistry, University of the Basque Country (UPV/EHU), Donostia-San Sebastián 20018, Spain

*Corresponding author: oihane.sanz@ehu.eus

Table S1. Main characteristics of the structured substrates used

	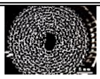	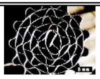	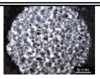	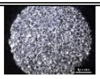	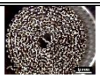	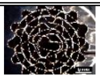	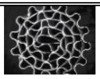	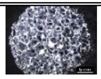
Name	MF_2360	MF_289	FF_40	FF_60	MB_2360	MB_289	MA_289	FA_40
Alloy	FeCrAl				Brass		Aluminum	
Dimensions Ø x L (mm)	16 x 30		16 x 25.4		16 x 30			
Cell or pore density	2360 cpsi[a]	289 cpsi[a]	40 ppi[b]	60 ppi[b]	2360 cpsi[a]	289 cpsi[a]	240 cpsi[a]	40 ppi[b]
Geometric surface area (m^{-1})	87.6	35.3	19.1[c]	26.4[c]	87.6	35.3	23.5	17.4[c]
Ke,a[d] (W/m·K)	4.0	1.6	-	-	28.8	11.5	22.0	-
Ke,r[d] (W/m·K)	3.4	1.9	0.6-0.9[e]	-	18.4	7.2	12.9	7.7-7.9[e]

[a] cpsi= cells per square inch; [b] ppi = pore per inch; [c] Manufacturer's information; [d] Axial ($k_{e,a}$) and radial ($k_{e,r}$) effective thermal conductivity calculated from ref.[1]; [e] Data from references[2,3]

Table S2. Physicochemical properties of structured catalysts with different substrates

Sample	Washcoating			N_2 Adsorption			N_2O-RFC	H_2-TPR	
	δ[a] (μm)	No. of coatings	Adh. (%)	S_{BET} (m^2/g)	V_{PORE} (cm^3/g)	D_{PORE} (nm)	S_{Cu} (m^2/g_{CZA})	H_2 consumption (cm^3/g_{CZA})	Reducibility (%)
Slurried catalyst	-	-	-	179	0.41	9.3	34.7	167	99
MF_2360_W1	19.9	11	91	180	0.48	10.6	31.2	181	107
MF_289_W1	49.4	18	89	183	0.49	10.7	29.8	176	104
FF_40_W1	91.6	13	91	174	0.46	10.5	28.8	166	98
FF_60_W1	66.2	9.3	85	193	0.49	10.2	30.3	180	106
MB_2360_W1	19.9	10	98	181	0.43	9.5	29.8	177	105
MB_289_W1	49.4	16	97	177	0.45	10.1	29.9	186	110
MA_289_W1	73.9	17	93	174	0.52	11.9	29.0	172	101
FA_40_W1	99.8	14	87	175	0.48	11. 0	29.8	167	99

[a] The average washcoat thickness was calculated from the geometric surface area of the structured substrates, the catalyst load and estimate of the coating density (0.989 g/cm^3).

Table S3. CO conversion and selectivity data, radial (R) and axial (A) temperature differences, and volumetric heat duty obtained in activity tests at 533 K and 4 MPa

Sample	Space velocity ($L_{syn}/g_{cat}\cdot h$)	X_{CO} (%)	Selectivity (%)				ΔT_R (ºC)[a]	ΔT_A (ºC)[b]	Q (kW/m^3)[c]
			MeOH	DME	CO_2	Others			
Slurried catalyst	1.7	81.3	3.0	70.6	26.3	0.1	-	-	78.0
MF_2360_W1	1.7	75.5	3.1	70.7	26.1	0.1	1	1	72.4
	3.4	52.7	3.1	70.6	26.2	0.1	1	0	101
	6.8	26.6	3.5	70.7	25.7	0.1	2	0	102
MF_289_W1	1.7	71.6	3.1	70.5	26.2	0.2	2	1	68.7
	3.4	52.2	3.3	70.3	26.3	0.1	2	0	100
	6.8	25.2	3.8	70.1	26.0	0.1	2	0	96.7
FF_40_W1	1.7	76.1	3.3	70.6	26.0	0.1	0	0	73.0
	3.4	55.7	3.2	70.5	26.2	0.1	0	1	107
	6.8	30.5	3.5	71.0	25.3	0.2	0	1	117
FF_60_W1	1.7	73.0	3.0	69.8	27.1	0.1	0	1	70.0
	3.4	51.3	3.0	69.5	27.4	0.1	0	1	98.4
	6.8	26.8	3.7	70.8	25.4	0.1	0	1	103
MB_2360_W1	1.7	71.9	2.9	70.7	26.3	0.1	2	1	69.0
	3.4	48.0	2.9	70.4	26.6	0.1	2	1	92.1
	6.8	24.0	3.3	71.7	24.9	0.1	2	1	92.1
MB_289_W1	0.8	87.4	3.6	70.8	25.5	0.1	1	1	39.5
	1.7	75.8	3.6	71.1	25.2	0.1	2	1	72.7
	3.4	54.3	4.2	70.8	24.9	0.1	2	1	104
	6.8	30.0	4.0	69.2	26.6	0.2	2	1	115
MA_289_W1	1.7	72.0	2.9	70.7	26.2	0.2	0	1	69.1
FA_40_W1	1.7	73.8	2.8	70.5	26.6	0.1	0	0	70.8

[a] ΔT_R is the difference between the readings of the thermocouple located at the monolith center and that on the outer edge of the monolith at the same reactor length.

[b] ΔT_A is measured between the reaction temperature control point, located at the exit of the monolith at r = 0, and the measurement point located at the monolith center.
[c] Q is the volumetric heat duty, the heat produced per total monolith volume

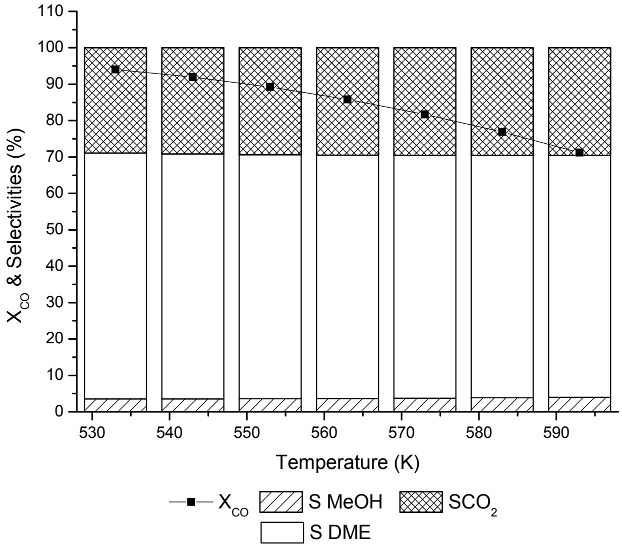

Figure S1. CO conversion and selectivities for direct synthesis of DME in the equilibrium at different temperatures.

References

1 Visconti, C.G.; Groppi, G.; Tronconi, E. Accurate prediction of the effective radial conductivity of highly conductive honeycomb monoliths with square channels. *Chem. Eng. J.* **2013,** *223,* 224.

2 Bianchi, E.; Heidig, T.; Visconti, C.G.; Groppi, G.; Freund, H.; Tronconi, E. Heat transfer properties of metal foam supports for structured catalysts: Wall heat transfer coefficient. *Catal. Today* **2013,** *216,* 121.

3 Bianchi, E.; Heidig, T.; Visconti, C.G.; Groppi, G.; Freund, H.; Tronconi, E. An appraisal of the heat transfer properties of metallic open-cell foams for strongly exo-/endo-thermic catalytic processes in tubular reactors. *Chem. Eng. J.* **2012,** *198-199,* 512.
